# Application of Artificial Intelligence Technology in Oncology: Towards the Establishment of Precision Medicine

**DOI:** 10.3390/cancers12123532

**Published:** 2020-11-26

**Authors:** Ryuji Hamamoto, Kruthi Suvarna, Masayoshi Yamada, Kazuma Kobayashi, Norio Shinkai, Mototaka Miyake, Masamichi Takahashi, Shunichi Jinnai, Ryo Shimoyama, Akira Sakai, Ken Takasawa, Amina Bolatkan, Kanto Shozu, Ai Dozen, Hidenori Machino, Satoshi Takahashi, Ken Asada, Masaaki Komatsu, Jun Sese, Syuzo Kaneko

**Affiliations:** 1 Division of Molecular Modification and Cancer Biology, National Cancer Center Research Institute, 5-1-1 Tsukiji, Chuo-ku, Tokyo 104-0045, Japan; masyamad@ncc.go.jp (M.Y.); kazumkob@ncc.go.jp (K.K.); norio.shinkai@riken.jp (N.S.); masataka@ncc.go.jp (M.T.); rshimoya@ncc.go.jp (R.S.); dr200004@tmd.ac.jp (A.S.); ktakazaw@ncc.go.jp (K.T.); abolatka@ncc.go.jp (A.B.); kshozu@ncc.go.jp (K.S.); ai.okky61@gmail.com (A.D.); hmachino@ncc.go.jp (H.M.); sing.monotonyflower@gmail.com (S.T.); ken.asada@riken.jp (K.A.); maskomat@ncc.go.jp (M.K.); sesejun@humanome.jp (J.S.); sykaneko@ncc.go.jp (S.K.); 2 Cancer Translational Research Team, RIKEN Center for Advanced Intelligence Project, 1-4-1 Nihonbashi, Chuo-ku, Tokyo 103-0027, Japan; 3 Department of NCC Cancer Science, Graduate School of Medical and Dental Sciences, Tokyo Medical and Dental University, 1-5-45 Yushima, Bunkyo-ku, Tokyo 113-8510, Japan; 4 Indian Institute of Technology Bombay, Powai, Mumbai 400 076, India; kruthisuvarna5@gmail.com; 5 Department of Endoscopy, National Cancer Center Hospital, 5-1-1 Tsukiji, Chuo-ku Tokyo 104-0045, Japan; 6 Department of Diagnostic Radiology, National Cancer Center Hospital, 5-1-1 Tsukiji, Chuo-ku, Tokyo 104-0045, Japan; mmiyake@ncc.go.jp; 7 Department of Neurosurgery and Neuro-Oncology, National Cancer Center Hospital, 5-1-1 Tsukiji, Chuo-ku, Tokyo 104-0045, Japan; 8 Department of Dermatologic Oncology, National Cancer Center Hospital, 5-1-1 Tsukiji, Chuo-ku, Tokyo 104-0045, Japan; sjinnai@ncc.go.jp; 9 Humanome Lab, 2-4-10 Tsukiji, Chuo-ku, Tokyo 104-0045, Japan

**Keywords:** machine learning, deep learning, artificial intelligence, precision medicine, radiology, pathology, omics

## Abstract

**Simple Summary:**

Artificial intelligence (AI) technology has been advancing rapidly in recent years and is being implemented in society. The medical field is no exception, and the clinical implementation of AI-equipped medical devices is steadily progressing. In particular, AI is expected to play an important role in realizing the current global trend of precision medicine. In this review, we introduce the history of AI as well as the state of the art of medical AI, focusing on the field of oncology. We also describe the current status of the use of AI for drug discovery in the oncology field. Furthermore, while AI has great potential, there are still many issues that need to be resolved; therefore, we would provide details on current medical AI problems and potential solutions.

**Abstract:**

In recent years, advances in artificial intelligence (AI) technology have led to the rapid clinical implementation of devices with AI technology in the medical field. More than 60 AI-equipped medical devices have already been approved by the Food and Drug Administration (FDA) in the United States, and the active introduction of AI technology is considered to be an inevitable trend in the future of medicine. In the field of oncology, clinical applications of medical devices using AI technology are already underway, mainly in radiology, and AI technology is expected to be positioned as an important core technology. In particular, “precision medicine,” a medical treatment that selects the most appropriate treatment for each patient based on a vast amount of medical data such as genome information, has become a worldwide trend; AI technology is expected to be utilized in the process of extracting truly useful information from a large amount of medical data and applying it to diagnosis and treatment. In this review, we would like to introduce the history of AI technology and the current state of medical AI, especially in the oncology field, as well as discuss the possibilities and challenges of AI technology in the medical field.

## 1. Introduction

The rapid progress in machine learning technologies, especially deep learning, along with the development of information infrastructure technologies such as the graphics processing unit (GPU), and the development of public databases, have made it possible to make use of large scale data called big data and have aroused a great deal of interest in artificial intelligence (AI) technology worldwide [1]. Historically, AI research has been conducted for a relatively long time, and the term “artificial intelligence” was already being used as an academic term by the 1950s [2]. However, AI research has not been a smooth road, and it has gone through a difficult period known as the AI winter period. The current boom is being dubbed the third AI boom [3], but this one differs from the previous booms in which many AI technologies in that it is being implemented in society. Face recognition technology based on AI technology is actively being used in airports and other areas, and AI is currently being used in various fields of society, including voice recognition, automatic translation, and automated driving. In fact, more than 60 medical devices with AI have been approved by the FDA in the United States, and the aggressive introduction of AI technology into the medical field in the future is inevitable. The field of oncology is no exception to this trend, and several AI-equipped medical devices are already being used for clinical applications, especially in radiology.

Current AI uses machine learning technology as the core technology [1]. Machine learning techniques are methods that learn from sample data, find patterns in these, and apply them to new data for analysis and prediction [4]. Whereas traditional statistics is often used primarily for explanatory purposes, machine learning uses it primarily for predictive purposes. There is a variety of machine learning techniques, which can be divided into two main types: supervised and unsupervised learning [5]. Supervised learning is a method of extracting features from given information to make predictions, which can be divided into classification and regression problems. The classification problem is used to predict discrete data, for example, distinguishing between benign and malignant tumors [4]. The regression problem is used to predict continuous data, for example, contrast effects. Unsupervised learning is a method of summarizing similarities for a given set of data on a problem for which no answer has been given. Because the classification problem of supervised learning is close to diagnosis, it is also considered to be the most familiar problem for clinicians, such as radiologists [4]. Deep learning technology is a part of machine learning technology; it currently holds great promise in the medical field. Deep learning techniques are used for medical image classification, image quality improvement, and segmentation because they are particularly good for image analysis. There are various types of deep learning techniques, and depending on the type of data to be handled, it is necessary to select the structure of the neural network that best suits the data [4]. Typical deep learning techniques used in medical imaging include convolutional neural networks for image classification [6], recurrent neural networks for reporting tasks [7], and a U-net for image segmentation [8].

In the field of oncology, the promotion of “precision medicine,” which refers to the medical care optimized for each patient by extracting important information from medical big data, especially genomic information, has become a global trend. “Precision oncology” and “precision cancer medicine” are also increasingly being used as academic terms [9,10,11,12,13]. However, the selection of treatment options based on a targeted-gene panel (TGP) coupled with next-generation sequencing, which is the core method of precision medicine, has become a major issue in promoting precision medicine because there is only a limited number of patients for whom the most appropriate treatment is suggested [14,15,16]. In order to increase the number of patients who may benefit from precision medicine, it is considered essential to extract pertinent information from additional medical data, including whole genome and epigenome information, in addition to limited genetic mutation information. However, the amount of information that needs processing has increased significantly; for example, whole genome analysis can yield up to 3 billion base pairs of information. To analyze these very large data efficiently and accurately, the use of cutting-edge AI and information and communications technology (ICT) technologies is required.

In this review, we introduced the history of AI technology and discussed how AI technology is currently being applied in the medical field and implemented in society, with a focus on the field of oncology. In addition, while AI technology has great potential, it is still associated with many issues that need to be resolved. We discussed the current issues in the field of medical AI and what kind of efforts are needed to continue to resolve these issues.

## 2. History of Artificial Intelligence and Its Application in Medicine

### 2.1. From the 18th Century to the 19th Century: Bayes’ Theorem and Pierre-Simon Laplace

In 1763, ”An Essay towards solving a Problem in the Doctrine of Chances,” including theorems of conditional probability which form the basis of Bayes’ Theorem, was published [17]. This work is based on the mathematical theory of probability by the Reverend Thomas Bayes, with multiple revisions and additions by his friend Richard Price, and was not published until 2 years after Thomas Bayes’ death. Meanwhile, the theory itself did not receive attention for a while after publication. In 1814, Pierre-Simon Laplace, a French scholar and polymath, set out a mathematical system of inductive reasoning based on probability as follows: where event *A_j_* ∈ {*A*_1_, *A*_2_, …, *A_n_*} exhausts the list of possible causes for event *B*, Pr(*B*) = Pr(*A*_1_, *A*_2_, …, *A_n_*). Then:

(1) $$\mathrm{Pr}(A_{i\;}|B)=\mathrm{Pr}\left(A_{i}\right)\frac{\mathrm{Pr}(B\;|A_{i})}{\sum_{j}\mathrm{Pr}\left(A_{j}\right)\mathrm{Pr}(B\;|A_{j})}$$

This formula is now widely known as Bayes’ Theorem. Pierre-Simon Laplace used what would now be considered Bayesian methods to solve many statistical problems, and a number of Bayesian methods have been developed by later authors. On the other hand, as the frequentist statisticians, represented by Karl Pearson, Ronald Fisher and Jerzy Neyman, came to be at the core of statistics, Bayesianism was considered unfavorable by many statisticians based on philosophical and practical considerations. As a result, the term “Bayesian method” was not commonly used to describe such a method until the 1950s. However, with the advent of powerful computers and new algorithms (especially the significant contribution of the Markov chain Monte Carlo method), Bayesian methods are still being used in statistics in the 21st century [18,19].

### 2.2. The Birth of AI, First AI Boom, and First Phase of the AI Winter

When computers became accessible in the mid-1950s, several scientists intuitively realized that two machines that could manipulate numbers could also manipulate symbols, and that the manipulation of symbols could represent the essence of human thought. This became a new way of approaching “thinking machines.” In 1956, Dr. John McCarthy proposed the name “Artificial Intelligence (AI)” at the Dartmouth Workshop, and the study of AI as an academic field was launched [2,20]. The years after the Dartmouth Conference were an era of discovery, and the momentum was such that computer scientists were galloping through new horizons. Programs developed in this era relied on reasoning and exploration, and even the best computers of the time, which were developed at great expense, could only solve problems in a very limited domain because the amount of computation they could handle was very small; however, they were still “astounding” to people at the time. The computers showed people how to solve algebra problems, prove geometry theorems, and learn to speak English [21]. However, in the 1970s, AI was subjected to criticism and shrinking funding. One reason was that the AI researchers failed to properly assess the difficulty of the problem they were facing. Expectations of the results were too optimistic and high; however, the results failed to meet these expectations, and funding for AI research largely disappeared.

### 2.3. The Second AI Boom and Second Phase of the AI Winter

In the 1980s, knowledge representation became a focus of AI research as “expert systems,” a form of AI program, were adopted by companies around the world [21,22,23]. An expert system is a program that answers questions and solves problems in a specific domain of knowledge and uses logical rules extracted from expert knowledge. Early examples include the Dendral (1965), which identifies compounds from spectrometer measurements and was developed by Dr. Edward Feigenbaum and colleagues [24], and MYCIN (1972), which diagnoses contagious blood diseases and was developed by Dr. Edward Shortliffe and colleagues [25]. MYCIN used a fairly simple reasoning engine and had a knowledge base consisting of about 500 rules [26]. This expert system asked the physician a number of questions that could be answered with a simple “yes/no” answer or some kind of written answer and finally gave a list of the names of bacteria that could be responsible (in order of probability), the confidence level of each, reasons why they were considered in the list, and the recommended course of drug therapy [27]. Despite its success, MYCIN is now sometimes regarded as an example of the risks of creating ad hoc probabilistic frameworks, such as in artificial intelligence lectures. One of the reasons for this is that MYCIN’s depth of inference was limited because of the noise introduced by the confidence coefficient system. This problem can be prevented by adopting a rigorous probabilistic framework, such as the Bayesian estimation [28].

The growing interest in AI in the 1980s was temporary and followed the classic pattern of the bubble economy. In the end, expert systems were only effective in very limited circumstances, and in the 1980s and early 1990s, AI research again ran into a funding crisis.

### 2.4. The Third AI Boom and Era of Deep Learning

In 2006, deep learning using auto-encoders was invented by the Dr. Jeffrey Hinton group [29]. This invention was a major breakthrough in AI because it could extract features without human intervention, eliminating the need for human knowledge representation. In addition, this invention also solved the problem of symbolic grounding, which was caused by human knowledge representation. In 2010, the term “big data” was proposed in response to the increasing amount of data transfer through the Internet. The 2012 ImageNet Large Scale Visual Recognition Competition (ILSVRC) competition for object recognition rates, which featured a major breakthrough in large-scale GPU-based deep learning (led by Dr. Jeffrey Hinton’s research team on Alex-net) [30], and Google’s announcement in the same year of its success in recognizing a cat from YouTube images using deep learning, led to a renewed interest in artificial intelligence research around the world [31]. AI technology is actively used in the medical field as well, and its effectiveness has been demonstrated in various results such as medical image analysis and omics analysis [1,32,33,34,35,36,37,38,39]. In the field of oncology, the introduction of AI is also being actively studied in tumor screening, including lung and breast cancer [40,41]. Practically, as of 2020, more than 60 AI-powered medical devices have been approved by the US FDA (Table 1). One important thing that sets the third AI boom apart from the previous AI booms is that it is actually being implemented in society. Dr. Demis Hassabis, the CEO and co-founder of DeepMind, mentioned that throughout the history of AI, we have repeatedly climbed up and down the wrong ladder, but it is important that we have finally arrived at the “right ladder.” Additionally, Bayesian statistics is also currently receiving a lot of attention due to the advances in AI technology. The reason for this is that in Bayesian statistics, the probability is set at the beginning, and subsequently the probability at that point of time can be changed as more information becomes available. This phenomenon itself resembles the “learning ability” of humans. It is this feature of Bayesian statistics that is the basis of deep learning and machine learning, which is an important feature of AI. 

## 3. Introducing AI Technology in Oncology

### 3.1. Radiology

**Figure 1:**
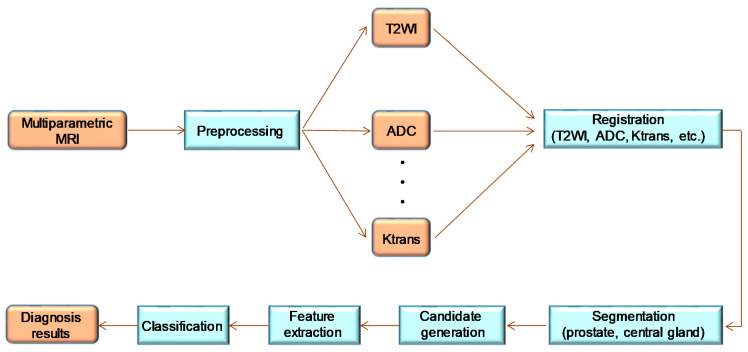
Workflow diagram of a typical prostate CADx system in Reference 44 (Wang et al., 2014); this is a modified figure from the reference. The orange squares indicate the data (original scan and pre-processed image), and the light blue squares indicate the data and processing applied to the image.

In terms of the medical applications of AI, radiology is one of the areas where AI technology has been maximally adopted [42]. In fact, most of the medical devices with AI approved by the FDA that are related to oncology are in the field of radiology (Table 1). The reason for this is that deep learning technology is prominent in image analysis, and radiation image analysis is one of the historically advanced ICT fields such as computer-assisted detection/diagnosis (CAD), which has a strong affinity for AI [43]. Figure 1 shows a typical CADx workflow for detecting prostate cancer [44]. In cancer diagnosis, CAD has been expected to improve productivity in image reading by improving the diagnostic accuracy and reproducibility of image reading, as well as reducing the reading time [45,46].

Approved by the FDA in May 2017, AmCAD-US (AmCad BioMed Corporation) is a software device that visualizes and quantifies the statistical distribution of backscattered signals echoed by tissue compositions in the body from ultrasound systems [47]. AmCad BioMed Corporation has also developed and received FDA approval for AmCAD-UT, primarily for the detection of thyroid cancer [48]. In July 2017, the Second Reader type CADx (QuantX) by Quantitative Insights received its first FDA approval [49]. This device is designed to target breast magnetic resonance imaging (MRI) and assist physicians in the differential diagnosis of breast cancer. In January 2018, Arterys Oncology DL (Arterys), which can help clinicians to rapidly measure and track lesions and nodules on MRI and computed tomography (CT) scans, received FDA approval [50]. This device was developed to support the diagnosis of solid tumors in general, and is in the first stage of clinical application for liver and lung cancer. In November 2018, SubtlePET (Subtle Medical), an AI-based high-speed positron emission tomography (PET) imaging technology, was approved by the FDA [51]. SubtlePet is an AI technology that enables four times faster imaging, especially during PET imaging, which plays an important role in cancer diagnosis. In March 2019, cmTriage (CureMetrix) was approved by the FDA for the triage of mammography images [52]. cmTriage is a workflow optimization tool that allows radiologists to customize, sort, and prioritize mammography worklists, allowing them to prioritize cases that require immediate attention. In addition, cmTriage can be used to optimize clinical workflows by sending suspicious cases to experienced radiologists or by immediately notifying them of suspicious cases even just before a patient is discharged. In April 2019, Deep Learning Image Reconstruction (DLIR: GE Medical Systems) was approved by the FDA for use as a specialized deep neural network (DNN) to generate TrueFidelity CT images [53]. TrueFidelity CT imaging has the potential to improve reading reliability in patients of all ages in a wide range of clinical applications, including head, systemic, and cardiovascular applications. In June 2019, the Advanced Intelligent Clear-IQ Engine (AiCE: Canon Medical Systems Corporation) was approved by the FDA [51]. AiCE is a state-of-the-art image reconstruction technique for CT designed using deep learning, which uses a process that discriminates between noise and signal components to selectively remove noise while maintaining spatial resolution. By using AiCE, a high noise reduction effect can be obtained while maximizing the spatial resolution of the CT scanner. In addition, a high noise reduction effect is achieved in low-contrast regions while maintaining the granularity, and stable image quality improvement is achieved even in low-dose regions. ProFound™ AI Software V2.1 (iCAD), approved by the FDA in October 2019, is a CAD software that assists physicians during digital breast tomosynthesis (DBT) [54]. The system is capable of detecting soft tissue density (masses, architectural distortions, and asymmetries) and calcifications within 3D DBT slices, with the aim of improving breast cancer detection rates and making a more accurate diagnosis. Approved by the FDA in December 2019, Transpara (ScreenPoint Medical) is an AI system that specializes in image interpretation in mammography [54]. Studies on the Transpara system showed that using this system to assist in image interpretation can significantly improve the area under the receiver operating characteristic curve (AUC) and diagnostic sensitivity without increasing the interpretation time. Additionally, the AUC of the Transpara system alone was comparable to the average AUC of radiologists.

### 3.2. Endoscopy

Endoscopic images are also an important target of AI analysis. In particular, given that Japanese medical equipment manufacturers hold a 99.1% share of the global market for endoscopes, endoscopic AI development is being actively conducted in Japan [55]. At present, there is an example of clinical implementation of endoscopic AI in Japan after receiving regulatory approval from the Pharmaceuticals and Medical Devices Agency (PMDA) [56]. We also developed a real-time endoscopic diagnostic support system for the detection of colorectal cancer (CRC) and precancerous lesions using AI technology in order to improve the lesion detection rate by colonoscopy, eliminate technology gaps among physicians, and prevent missed lesions [36]. When developing endoscopic AI, we planned to first target CRC; one of the reasons was its high frequency. The latest data show that CRC affects about 130,000 people in Japan annually, and the number of deaths exceeds 50,000 per year [57]. Additionally, the number continues to rise. Meanwhile, CRC and other cancers of the gastrointestinal tract can be controlled if they are detected early with endoscopic intervention. In fact, in the United States, the results of the National Polyp Study reported in 1993 and its cohort study reported in 2012 demonstrated that the endoscopic removal of adenomatous polyps, a precursor lesion to CRC, reduced the morbidity of CRC by 76–90% and mortality by 53% [58,59]. Therefore, endoscopy is actively used for physical examination as well as gastric and colorectal cancer screening, and when adenomatous polyps are found in the colon, they are removed endoscopically. In other words, two things are important for the prevention of CRC: (1) undergoing a colonoscopy; and (2) not missing a lesion during the examination. Interventions to increase the uptake of countermeasure screening are necessary to promote colonoscopy. However, the process from colonoscopy to polypectomy consists of five major stages: 1. lesion detection, 2. qualitative and quantitative diagnosis, 3. treatment, 4. pathological diagnosis, and 5. surveillance. This means that the first thing to do is not to miss any lesions during the examination. The endoscopist can easily detect the so-called “polyps” if they are protruding in shape; however, flat-shaped lesions or lesions that are the same color tone as the surrounding mucosa are less visible and require a certain amount of experience for detection. In addition, the colon has many anatomical blind spots and requires techniques to observe the lumen in every corner of the colon, including between the mucosal folds in the wall. For these reasons, a colonoscopy is a test in which there is a technical gap on the part of the physician, which can be a major source of concern for the patient. Using the latest technology of deep learning, the Department of Endoscopy at the National Cancer Center’s Central Hospital trained AI on a large number of endoscopic images of early-stage colorectal cancer and precancerous lesions accumulated to date. In order to study a large number of unbiased cases, we first collected at least one still image of each consecutive early-stage colorectal cancer and precancerous lesion over a period of time. The location of the lesions in the images was then checked, and a line was drawn at the boundary between the surrounding mucosa and each lesion with a marker for all lesions, thus surrounding the lesions. Although it might seem to be a simple and steady task, it was important that there were no mistakes during this work because these data were used as training data for deep learning. We used our own convolutional neural network as a deep learning technique [36]. In conventional machine learning, the extraction of the lesion features in images is done manually by humans, but in deep learning, AI automatically generates a variety of lesion features. The input images are extracted as features in the convolution and pooling layers and their classification is presented in the output layer. Moreover, the AI automatically learns from the training data using the back-propagation method. A performance evaluation test on the developed AI system was performed on 705 images with lesions and 4135 images without lesions obtained at our hospital over a period of time that were not used for training. For the evaluation study, the researchers involved in the analysis of the AI were completely blind to lesion and clinical information. The overall sensitivity and specificity of the AI for colorectal lesions were 98.8 and 99%, respectively, and a correct answer was considered to be correct if the AI correctly detected the location of the lesion. Furthermore, we succeeded in analyzing a single frame of video (lesion detection and result display) within 33 milliseconds (30 frames per second). Therefore, we believe that the system has reached a stage where it can be developed for clinical implementation as an endoscopic diagnostic support system [36].

### 3.3. Pathological Images

Pathological diagnosis is the final diagnosis of a lesion and therefore plays an important role in determining the subsequent treatment strategy and the effectiveness of treatment. However, there is currently a shortage of pathologists in the United States, United Kingdom, Japan, Canada, and other countries around the world, and this has become a problem for maintaining the quality of medical care in each country [60,61]. Under these circumstances, research and development in pathological diagnosis using AI technology is of paramount importance. However, there are some problems that need to be solved, such as how to standardize the pathological images, because the methods for preparing pathological specimens and staining methods vary from institution to institution. Therefore, pathological diagnosis using AI technology has not yet been actively introduced into clinical practice. However, there is no doubt that the utilization of AI for pathological diagnosis is one way to compensate for the shortage of pathologists, and it is likely that pathological diagnosis using AI technology will gradually advance in clinical practice in the future. An important finding on pathological diagnosis using AI technology was recently published by New York University, USA [62]. In this study, Coudray et al. trained a large number of high-definition digitally imaged pathology glass slide specimen images (also known as Virtual Slides) using a deep learning algorithm called InceptionV3 for histopathological classification (lung cancer (adenocarcinoma and squamous cell carcinoma) and normal lung). The results revealed a very high accuracy with 0.97 AUC for tissue classification [62]. Both frozen and formalin-fixed paraffin-embedded sections were available for analysis as specimens. Furthermore, using the developed AI analysis system, six gene mutations, STK11, EGFR, FAT1, SETBP1, KRAS, and TP53, could be accurately predicted from the pathological images (AUC: 0.733–0.856). These results suggest that the analysis of pathological virtual slide images using AI technology may enable the accurate classification of lung cancer tissues and prediction of genetic mutations. As a next step, it is important to conduct prospective studies to ensure the robustness of results across institutions; however, this is not limited to pathological image analysis, and we believe that this should apply to all medical image analysis systems that utilize AI technology.

### 3.4. Skin Images

Skin cancer is the most common malignancy in Western countries, with melanoma in particular accounting for the majority of skin cancer-related deaths worldwide [63]. Melanoma is a highly malignant skin cancer of melanocyte origin that is similar in shape to a mole; therefore, it is very important to distinguish between moles (benign) and melanomas (malignant). The early detection of skin cancer, including melanoma, is particularly crucial because the early detection of skin cancer makes it easier to treat and significantly improves prognosis [64]. However, melanoma is sometimes difficult to diagnose clinically because of its similarity to other brown to black pigmented lesions such as pigmented nevi, seborrheic keratosis, and basal cell carcinoma. Consequently, we developed a skin tumor determination system for brown to black pigmented lesions [33]. In this study, out of the data of 120,000 images of patients seen at our hospital from 2001 to 2017, we extracted images of brown to black pigmented skin lesions (malignant melanoma and basal cell carcinoma for malignant tumors, nevus pigmentosus, senile pigmentation, seborrheic keratosis, hematoma, and hemangioma for benign tumors), which were the diseases to be analyzed. After the extraction, each image was annotated by a dermatologist with a bounding-box to indicate the location in the image where the lesion was located. We then applied general object detection techniques to train a neural network to predict the location and type of lesion in the image. At the end of the training, the AI predicted the type of lesion for validation data (images not used for training), while clinicians (10 dermatologists and 10 non-specialists) were also tested using the same images. The results showed that the discrimination accuracy of the AI was comparable to that of dermatologists as well as non-specialists. Our goal is to make our skin tumor determination system available to the public so that users can become more aware of their own symptoms and skin tumors in general. Additionally, we hope to establish a framework that encourages them to seek medical attention when necessary. This can help reduce the proportion of patients with advanced stages of the disease and reduce the rising cost of healthcare.

Esteva et al. also attempted to classify skin diseases using AI by analyzing a database of about 130,000 images and their corresponding 2000 diseases, using one of the deep learning algorithms, convolutional neural network [65]. In this study, 127,463 images were trained and validated, and 1942 images were used to compare the diagnoses by AI and dermatologists. The results showed that the AUC values of 135 skin cancer cases and of 130 melanoma cases diagnosed by AI were 0.96 and 0.94, which were almost the same as that of the diagnosed by dermatologists [65].

## 4. Application of Machine Learning and Deep Learning Techniques to Omics Analysis

With the completion of the International Human Genome Project in 2003 [66], we entered an era known as the post-genome era, and the momentum for the application of genomic information in medicine has increased. As a result, the term “genomic medicine” has emerged, proposing a new type of medical treatment that provides patients with optimal treatment options based on the genome information [67]. In 2015, the Precision Medicine Initiative was announced by US President Barack Obama for cancer and rare diseases, dividing patients/potential sufferers into subgroups of disease morbidity and developing appropriate treatments and prevention methods for each group [68]. This initiative had a great impact on the global medical policy, and since then, the establishment of precision medicine has become one of the major goals of research in the field of oncology. However, the current state of precision medicine has many problems that need to be addressed. One of the main problems is that although the provision of optimal anticancer drugs based on TGP is the current mainstay of precision medicine, the number of patients who receive the most appropriate treatment is low [14,15,16]. Increasing the number of patients who benefit from precision medicine is considered an important issue; however, it is believed that treatment selection based on the current TGPs alone is insufficient to address this issue [15]. It is essential to integrate whole genome sequencing (WGS) data as well as other omics data, such as epigenomic, proteomic, and metabolomic data, for multimodal analysis. In particular, recent rapid advances in epigenomic analysis techniques have revealed that cancer cells accumulate a variety of epigenomic abnormalities in addition to genetic abnormalities [69,70,71,72,73,74,75,76,77,78,79,80,81,82]. Epigenomic aberrations have a significant impact on the characteristics of cancer from the early stages of development to developmental progression [83,84,85,86,87,88,89,90,91,92,93,94,95,96,97,98,99]. In addition, owing to development and progress in medical epigenetics, which targets epigenome abnormalities, it is important to analyze the pathogenesis of cancer with an appropriate consideration of epigenetic information [100,101,102,103,104].

As mentioned above, in order to promote precision medicine, it is necessary to take into account omics information such as epigenomic information and WGS information in addition to genomic information obtained by panel inspection, and this is a huge amount of data. In order to efficiently and accurately analyze this vast amount of medical big data, that is, in a sense, beyond human capabilities, it is essential to utilize the most advanced AI and ICT technologies. In particular, as we have previously mentioned, machine learning and deep learning technologies have the following four features, and are expected to play an important role in the multimodal analysis of medical big data [1]:

- Multimodal learning: different types of medical data (e.g., genomic, epigenomic, and proteomic data) can be integrated and treated as inputs;
- Multitasking learning: multiple different tasks can be learned simultaneously by sharing parts of the model;
- Representational and semi-supervised learning: acquire a way to represent data from large amounts of unlabeled data, making it possible to learn from small amounts of labeled data;
- It is possible to capture higher order correlations of inputs.

**Figure 2:**
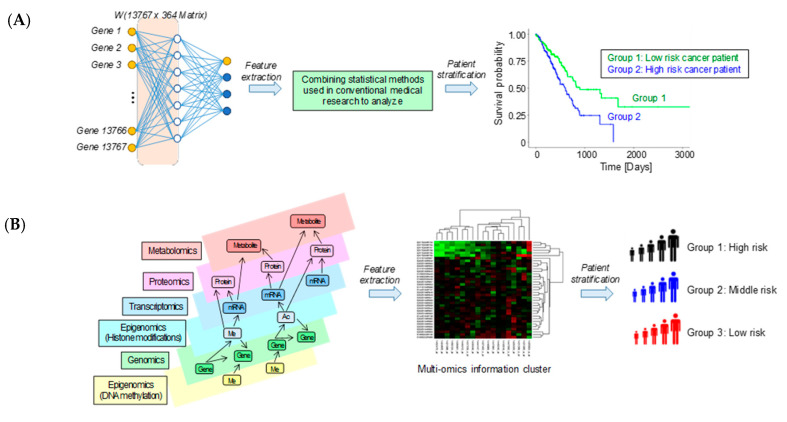
Examples of how machine learning and deep learning techniques are being used to reduce the dimensionality of massive omics data to extract features and then stratify the patients. (**A**) An example of analysis using Kaplan–Meier curves after dimensionality reduction and feature concentration using machine learning and deep learning techniques applied to genetic mutation information. (**B**) An example of patient stratification using machine learning techniques to extract features from multilayer omics data.

The above properties have led to the introduction of machine learning and deep learning techniques in medical large data analysis. In particular, machine learning and deep learning techniques are being widely used for dimensionality reduction and feature extraction in the stage of extracting important information from vast amounts of data, as well as for the stratification of patients based on the extracted features (Figure 2). For example, a method for dimensionality reduction using an autoencoder has been proposed for large amounts of multi-omics data [105], and we have also used this method to stratify lung cancer patients [32]. In this study, we combined RNA-seq and miRNA expression data from The Cancer Genome Atlas (TCGA), while focusing on lung adenocarcinoma (LUAD) with clinical information and performed a multi-omics analysis using an autoencoder. Consequently, we successfully subclassified patients according to survival (categorizing good and poor lung cancer prognosis groups). The classifier was developed using estimated labels derived from patient subtypes, the support vector machine (SVM) gave the best classification results, with an accuracy of 0.82 for the test data set. These subtypes were used to rank genes based on their RNA expression levels. The top 25 genes were investigated to identify the mechanisms that influence patient prognosis. Bioinformatics analysis showed that the expression levels of six of the 25 genes (*ERO1B*, *DPY19L1*, *NCAM1*, *RET*, *MARCH1*, and *SLC7A8*) were associated with survival in LUAD patients, and pathway analysis indicated that major cancer signaling pathways were altered in the subtypes. Expanding on this method, we identified survival-related subtypes of non-small cell lung cancer from six categories of TCGA multi-omics datasets (miRNA, mRNA, DNA methylation, somatic mutation, copy number variation, and reverse phase protein array) [106]. As a result, the subtype named the Integrated Survival Subtype, which combined the six types of data, successfully separated the poor and good prognosis groups of lung cancer patients with a statistically significant difference. We also confirmed that this was independent of the histopathological classification. In addition, the predicted subtypes were able to distinguish between high- and low-risk patients. Through the above studies, we presented a new potential multi-omics analysis to accurately predict the prognosis of lung cancer patients.

There are also several challenges in the application of deep learning for medical omics data, such as genomic data. First, the dimensions of the input are in orders of magnitude larger than the number of samples (Large *p*, Small *n* Problems) [107], and the model tends to overfit the training dataset. Second, the contribution of each input variable to the prediction is usually difficult to interpret because of the multiple nonlinear operations. Third, genetic data may not be characterized by an innate structure. To mitigate these problems, we proposed a genomic analysis method using a modified diet network by adding per-element input scaling [34]. The original concept of Diet Networks, with transposed data matrices as inputs to the auxiliary network, can significantly reduce the number of parameters in a fully connected layer [108]. The effectiveness of our proposed method was assessed in a binary classification task based on the somatic mutation profile of lung cancer histopathology, i.e., adenocarcinoma and squamous cell carcinoma [34]. The dataset consisted of 950 cases and was subjected to 5-fold cross-validation to assess the performance of the model. The results showed that the prediction accuracy was about 80%, and the addition of per-element input scaling contributed significantly to the stability of the learning process [34]. The latent representation obtained inside the model allowed us to interpret and predict relationships between somatic mutation sites.

For the realization of precision medicine, predicting the effects of anticancer drugs is a vital issue, and attempts have been made to predict the effects of large-scale omics data using machine learning and deep learning techniques [109,110,111,112,113,114]. Shukla et al. analyzed the chromosome arm aneuploidy (CAA) profiles of 23,427 tumors to identify the aspects of tumor evolution, including the order in which CAAs can occur and the CAAs that can predict tissue-specific metastases [115]. In this study, the authors used machine learning techniques (deep neural network models) to identify 31 CAAs that powerfully altered the response to 56 chemotherapy drugs in cell lines representing 17 cancer types. The authors also found 1024 potentially lethal drug interactions. Notably, CAAs significantly outperformed mutations and local deletions/amplifications combined in predicting the drug response [115]. Thus, CAAs have the potential to predict cancer prognosis, shape tumor evolution, metastasis, and drug response and thus advance precision oncology.

The Bruton tyrosine kinase inhibitor ibrutinib is an effective treatment for patients with chronic lymphocytic leukemia (CLL); however, there is extensive heterogeneity in this disease. Rendeiro et al. attempted to predict the response to ibrutinib treatment by analyzing multi-layered omics data (immunophenotyping, single-cell RNA-seq, and ATAC-seq) together with clinical information using machine learning techniques [116]. Non-malignant B cell changes reflected changes in the CLL cells, with CD4+ T cells, CD8+ T cells, natural killer (NK) cells, and myeloid cells responding in a cell type-specific manner [116]. They also identified a gene expression signature that captures the ibrutinib-induced widespread downregulation of immune cell function and the acquisition of a quiescent state in response to ibrutinib therapy. There was patient-specific variability in the speed of execution of the program, and this variability could be used to predict patient-specific dynamics of the response to ibrutinib based on the pre-treatment patient sample [116]. This study revealed time-dependent cellular, molecular, and regulatory effects on the therapeutic inhibition of B-cell receptor signaling in CLL using machine learning multi-omics analysis and established a widely applicable method for epigenomic/transcriptomic-based therapeutic monitoring.

Recently, the establishment of precision oncology based on large-scale omics analysis using machine learning and deep learning techniques has been actively studied; however, the research is still in its infancy. One of the current problems is that the biological properties of a gene mutation vary depending on the function of the gene and the location of the mutation, but we have not been able to take such detailed information into account, and all the mutations are lumped together for analysis. In the future, we need to consider methods to effectively introduce this kind of domain knowledge into the analysis. In addition, omics information, such as genomic and epigenomic information, has a large number of parameters compared to the number of samples, which is always a problem (Large *p*, Small *n* Problems) [107]. This makes it difficult to analyze the raw data as it is, and dimensionality reduction is necessary. An important point is the need to select a model that allows the compression of parameters without compromising the expressive power of the model; therefore, further study is needed. The explanatory and interpretive nature of the results also requires further exploration and is discussed in detail in Section 6.2. Furthermore, recent studies have identified important molecular mechanisms/signaling pathways in cancer development and progression [71,117,118,119,120,121,122,123,124,125,126,127,128,129,130,131,132,133,134,135,136,137,138], and several pathway analysis methods have been reported to elucidate the true nature of cancer and identify drug targets by using features extracted from large-scale data. The methodology is correct, and several results have been published that have contributed greatly to the development of the field of oncology [139,140,141,142,143,144,145,146,147,148,149,150]. However, it should be adequately recognized that there are limitations to the results obtained by a dry lab approach, and it is important to validate the results obtained by the dry lab approach using appropriate wet lab experiments (cell-level studies or animal-level studies using mice). By feeding back the results of the wet lab experiments to the dry lab approach, it is expected that the accuracy of the results of the dry lab approach itself can be improved. This can be said of AI in general, and no matter how much progress AI makes in the future, it is very risky for humans to rely on AI’s judgment for everything. We believe that there should always be a human verification step. Ideally, we should have an accurate understanding of the strengths and weaknesses of humans and AI, making it possible to learn together and complement each other.

## 5. Drug Development Using Machine Learning and Bayesian Statistics in Oncology

**Figure 3:**
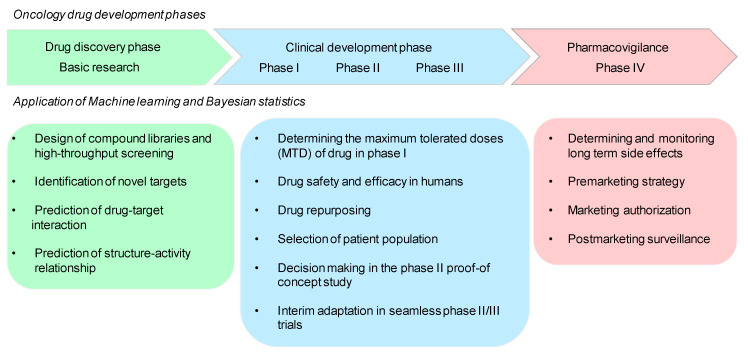
Application of machine learning and Bayesian statistics in different phases of drug development.

Drug development is a costly and time-consuming process that can last up to 15 years. The development pipeline starts with the initial phase 0, comprising basic research or drug discovery. The next three stages (phase I, phase II, and phase III) are clinical trials, while phase IV includes a pharmacovigilance study. Phase I involves the study of dose-toxicity and short-term side effects; the determination of drug performance occurs in phase II and phase III and involves comparing the drug to standard therapies for the disease being studied. Phase IV is to monitor the long-lasting side effects of the drug. The major challenge in the drug development process is the high failure rate and consequent financial loss in the final stages of development [151]. With the recent advances in AI platforms and machine learning techniques, it is now possible to fasten the pace of development as well as reduce the likelihood of failure. The machine learning models such as support vector machine, random forest, Bayes’ theorem, and many others find application in all the stages of drug development leading to accurate prediction and insights (Figure 3) [152]. The Bayesian approach is an emerging technique used by medical researchers in the field of oncology drug development. The issues of case fatality, survival analysis, dropouts from clinical observations, and complex computational problems can be effortlessly handled using Bayesian techniques. In the era of big data, the Bayesian statistical approach is better suited for combining the current data with prior knowledge and for creating posterior probabilities for both drug effectiveness and its safety [153].

The mathematical method using Bayesian statistics can be implemented at the design stage, during the conduct of the trial, at the analysis stage, for post-marketing surveillance purposes, and in meta-analysis. Recently, Bayesian Analysis to determine Drug Interaction Targets (BANDIT), an integrative big data approach, was developed for drug target prediction, validation for clinical development, and drug repurposing. This machine learning algorithm identified a novel microtubule inhibitor with activity against breast cancer cells that were resistant to all other clinically approved anti-microtubule drugs [154]. The Bayesian adaptive design can also be applied in phase I oncology trials, which are conducted in a small number of patients to determine the maximum tolerated dose (MTD) of the drug molecule [155]. A multicentered and non-randomized Bayesian adaptive design study on the γ-secretase inhibitor MK-0752 in combination with gemcitabine was conducted in patients with pancreatic ductal adenocarcinoma and successfully determined the safety of the combination treatment as well as identified the recommended dose for the phase II trial [156]. Yan et al. proposed an intuitive Bayesian keyboard decision method, which relies on the posterior distribution of the toxicity probability and can identify the true MTD with high accuracy [157]. The development of the oncology drugs usually involves a proof-of-concept study (PoC) at the end of the phase I or phase II trial. The PoC study is carried out to obtain an early evidence of the clinical efficacy using a small number of patients. Using a Bayesian framework, the decision making in PoC can be more effective since the direct estimation of evidence is possible for the effect of interest [158]. The Bayesian design can also shorten the duration of a cancer clinical trial by integrating the phase II/III trials into a single confirmatory study. Recently, ComPAS, a novel adaptive shrinkage method was developed using Bayesian model selection and hierarchical methods. This model allows for the dropping of ineffective drugs and the addition of new combinations to ongoing clinical pipelines based on accumulating trial data in an adaptive and seamless fashion [159].

The flexibility of the Bayesian statistics also makes it suitable for the network meta-analysis of pooled data that allows for the simultaneous comparison of multiple treatments. The characteristics of such pooled data are random from trial to trial, differing in the size of the trial, study design, and methodology. The random effects are best handled using Bayesian statistics, which can help to address unanswered questions from controversial clinical trials. The Bayesian network meta-analysis has been applied to assess the role of immunotherapies and targeted therapies in advanced melanoma. This model compared the therapies using the hazard ratio for the overall survival and progression-free survival and the odds ratio for the response rate and probabilities of the drug outperforming others. This meta-analysis suggested that combined BRAF-MEK targeted therapy is optimal for BRAF-mutant patients and can enhance the favorable outcomes in advanced melanoma [160].

Although the Bayesian method has been applied effectively in all phases of drug development in oncology, it is associated with several challenges. The application of the Bayesian method requires decision making regarding prior information, information obtained from the trial and the mathematical model to be used, at the initial design stage itself. A change in the prior information and the quality of the data at a later stage might affect the scientific validity of the trial results. It has been suggested that the type of statistical analysis used in cancer clinical trials should be determined at an earlier stage. The Bayesian adaptive design may suffer from operational biases; thus, the confidentiality of the data needs to be maintained [161]. The recent advances in machine learning algorithms and computational speed have made it possible to carry out calculations for complex Bayesian models. Furthermore, the integration of machine learning methods and statistical tools in drug development pipelines might decrease the cost and time of drug development and also enhance the development of precision medicine for cancer treatment.

## 6. Issues to Be Overcome in the Application of AI to Oncology

The importance of AI technology has been recognized worldwide, and several countries are promoting AI research as a national policy. Considering its great potential, there are high expectations from AI technology, and it is likely that AI technology will be increasingly introduced in the oncology field in the future. Despite the great potential of AI technology, there is still a number of challenges that need to be overcome. Therefore, we described the key challenges that need to be overcome on an ongoing basis.

### 6.1. Overfitting

In machine learning and deep learning techniques, overfitting refers to a situation where the training error is small, but the generalization error (the error in determining unknown data) is not small. Particularly in the medical field, where the amount of training data is limited, it is always necessary to carefully judge the generalization performance of the constructed model. We feel that validation is especially important when aiming for the clinical implementation of medical devices with AI; we need to confirm the general performance of these devices through clinical trials more carefully than we have done with conventional medical devices.

### 6.2. Black Box Problem

Since the analysis process of the machine learning and deep learning techniques is very complex, a black box problem arises in that humans cannot understand the analysis process of the results obtained. The presence of a black box in the system makes it difficult for the designer or user to predict the behavior of the system at the time of design or use and hinders the safety of the system. In Europe, the General Data Protection Regulation (GDPR), which came into force in May 2018, included an article (Article 22) requiring the transparency of AI; consequently, it is necessary to address the black box issue in terms of GDPR regulation compliance [162]. The following three approaches are mainly implemented to improve the interpretability of machine learning and deep learning.

Deep explanation: deep learning state analysis to generate attention heat maps and natural language explanations [163,164,165].

Interpretable models: machine learning using originally interpretable models (to improve the accuracy of white-box machine learning) [166,167].

Model induction: Create an interpretive model externally that approximates the behavior of black-box machine learning [168,169,170].

### 6.3. Discrepancies among Facilities, especially in Medical Imaging (Domain Shift and Domain Adaptation)

Medical imaging analysis is prone to facility characteristics (e.g., different manufacturers of devices, different model numbers of devices, differences in protocols, and differences in operators), and, we along with others, have observed in various studies that the accuracy of predicting data from other facilities is significantly reduced when a trainer built by training on data from one facility is used to predict data from another facility. In general, this problem is called the domain shift problem, and it is an important issue that needs to be resolved for the promotion of medical AI [171,172,173,174]. As an important study, we present the results of the analysis and validation of a large number of chest X-ray images using deep learning techniques, published by Pooch et al. [175]. They attempted to evaluate models trained independently on each of A, B, and C, using three large datasets from the U.S. National Institutes of Health (A: 112,120 images), Stanford University Hospital (B: 224,316 images), and the Massachusetts Institute of Technology (C: 379,920 images), with all of the A, B, and C data. When the data were evaluated by splitting the data into 80% training (10% of which were validated) and 20% testing, it was found that the models trained with the data from the same facility as the test data were more accurate, whereas the models trained with data from a different facility were found to be less accurate [175]. The results of this study show that even with such a large set of data, the generalization performance may not be properly evaluated owing to overfitting for each of the datasets. This can be attributed to the fact that the learning sets are independent of each other due to domain shifts. In other words, it was suggested that the model trained with data from each facility was overestimated when tested only with data from the same facility.

Domain adaptation has been proposed as one of the methods to solve the domain shift problem and is being actively studied [172,176,177,178,179,180,181,182,183,184,185,186,187]. Domain adaptation is a type of transfer learning in which knowledge obtained from a domain with sufficient labeled training data (source domain) is applied to a target domain that lacks sufficient information (target domain) to learn things such as discriminators that work with high accuracy in the target domain (here, a domain is a collection of data). There are several contexts of domain adaptation, and it is necessary to select an appropriate method for the target task.

Unsupervised domain adaptation: the training sample includes a set of labeled source examples, a set of unlabeled source examples, and a set of unlabeled target examples.

Semi-supervised domain adaptation: we also consider a “small” set of labeled target examples.

Supervised domain adaptation: all examples considered are assumed to be labeled.

**Figure 4:**
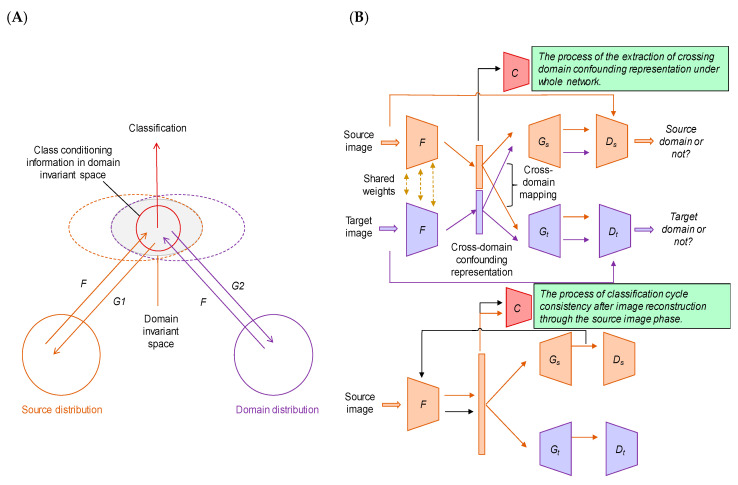
Illustration of the proposed network in Reference 188 (Qin et al., 2020); this is a modified figure from the reference. (**A**) Illustration of the proposed method: a cross-domain confounding representation is generated by constraining the cross-domain mapping reconstruction. (**B**) Domain confounding representation through cross-domain mapping and classification cycle consistency. Encoder *F* and decoder *G* constitute the variational automatic encoder (VAE) architecture for unsupervised representation learning. The *D* module constitutes the GAN discriminator and the *C* module constitutes the classifier.

As an example of the application of domain adaptation to medical image analysis, Qin et al. recently published an interesting study on the multi-center computer-assisted diagnosis of lymph nodes using unsupervised domain adaptation networks based on cross-domain confounding representation (Figure 4) [188]. In general, in order to achieve a robust, high performance computer-aided diagnostic system for lymph nodes, CT images may be collected from multicenter data, which can lead to model isolation based on different data source centers (the domain shift problem described above). However, the lymph node data variation adaptation problem related to the domain adaptation problem in deep learning is different from the general domain adaptation problem because the size of CT images is typically larger and the data distribution is more complex. Therefore, domain adaptation for this problem needs to take into account the shared feature representation of each region, as well as the conditioning information, so that the adaptive network can capture a significant discriminative representation in the domain invariant space. In this study, the authors extracted domain-invariant features based on cross-domain confounding representations, and proposed a cycle-consistency learning framework that encourages the network to retain class conditioning information through cross-domain image translations [188]. Figure 4A shows a conceptual diagram of the method adopted in this study. This method provided a better and more stable performance than did the conventional domain adaptation methods such as gradient reversal layer [189], maximum mean discrepancy [190], and Generate To Adapt [191] for high-resolution medical images with complex feature distribution. Figure 4B shows the process of extracting the cross-region confounding representation of the entire network and the classification cycle consistency after image reconstruction through the source image phase. Compared with the performance of different domain adaptation methods, this method achieved at least 4.4 percentage points higher accuracy for multicenter lymph node data. This method enables the stable domain adaptation of high-resolution images in complex medical fields. Experimental results on simple data distributions also show the generality of the proposed method. Furthermore, the stability of the learning process makes it possible to easily obtain the optimal model under the target domain, which may further realize the integration of multidisciplinary medical data.

Judging from the concept of Bayesian statistics, which is to make predictions based on the prior distribution of probabilities, it can be concluded that having a single algorithm with generalized performance for events with different prior distributions of probabilities from one facility to another is in itself a difficult task. Since AI technology is different from other technologies in that it has the ability to learn on its own, we believe that it is necessary to re-learn and optimize the training data, including the characteristics of each facility, in order to increase the potential of AI as much as possible. In this case, because medical devices themselves continue to evolve, which is a characteristic that conventional medical devices do not have, a multifaceted study that includes not only research and development but also the formulation of guidelines and legislation is necessary.

## 7. Concluding Remarks and Future Perspectives

In this review article, we described the application of AI technologies in the field of oncology, focusing on machine learning and deep learning technologies. Given that more than 60 types of AI-equipped medical devices have already been approved by the FDA, we believe that AI technology will be used as a core technology in the field of oncology and that the clinical implementation of this technology will steadily increase. Compared with medical image analysis, the introduction of machine learning and deep learning technologies for omics analysis, such as genomics and epigenomics, has lagged behind. However, with the realization of precision medicine as a goal all over the world, the utilization of AI technologies for omics analysis is expected to progressively rise in the future. Furthermore, as we have mentioned in this review, the introduction of AI in drug discovery is an important direction. Nevertheless, as mentioned previously, AI technology still has many problems that need to be addressed. Accordingly, it is important not to have excessive expectations of AI technology but to always calmly and objectively understand the advantages and disadvantages of the technology and steadily apply it to medicine. If humans become dependent on AI as a result of the development of AI, it will not lead to the establishment of an ideal human society. We believe that the ideal situation is for humans and AI to work together to improve the quality of cancer treatment and research.

## Figures and Tables

**Table 1:** List of AI-equipped medical devices approved by the US FDA.

No.	FDA Approval Number	Product Name (Company)	Description	Regulation Medical Specialty	Decision Date	Regulatory Class (Submission Type)
1	K140933	AliveCor Heart Monitor (AliveCor)	An ECG recording device using machine learning techniques to detect abnormal heart rhythms.	Cardiovascular	08/15/2014	Class II (510(k))
2	K143468	QbCheck (Qbtech)	A non-invasive test using AI for diagnosis and treatment of ADHD in children.	Neurology	03/22/2016	Unclassified (510(k))
3	K160016	Steth IO (StratoScientific)	An acoustic device using AI to aid in the identification of abnormal heart and lung sounds.	Cardiovascular	07/15/2016	Class II (510(k))
4	K163253	Arterys Cardio DL (Arterys)	A software using deep learning to visualize and quantify cardiovascular MR images.	Radiology	01/05/2017	Class II (510(k))
5	K161328	CANTAB Mobile (Cambridge Cognition)	An iPad-based memory-assessment tool for older adults.	Neurology	01/13/2017	Class II (510(k))
6	K162627	EnsoSleep (EnsoData)	An AI sleep scoring and analysis solution that automates event detection during sleep.	Neurology	03/31/2017	Class II (510(k))
7 *	K162574	AmCAD-US (AmCad BioMed Corporation)	A software to visualize and quantify ultrasound image data with backscattered signals.	Radiology	05/30/2017	Class II (510(k))
8 *	DEN170022	QuantX (Quantitative Insights)	An AI-equipped diagnosis system to aid in accurate diagnosis of breast cancer.	Radiology	07/19/2017	Class II (De Novo)
9	K172311	BioFlux Device (Biotricity)	A remote patient monitoring platform with AI.	Cardiovascular	12/15/2017	Class II (510(k))
10	K171056	WAVE Clinical Platform (Excel Medical Electronics)	A patient surveillance and predictive algorithm platform using AI.	Cardiovascular	01/04/2018	Class II (510(k))
11 *	K173542	Arterys Oncology DL (Arterys)	An AI-based, cloud-based medical imaging software that automatically measures and tracks lesions and nodules in MRI and CT scans.	Radiology	01/25/2018	Class II (510(k))
12	DEN170073	ContaCT (Viz.AI)	An AI algorithm to analyze CT scans and detect signs of stroke.	Radiology	02/13/2018	Class II (De Novo)
13	K170540	DM-Density (Densitas)	A machine learning application that provides on demand automated breast density assessments at point-of-care.	Radiology	02/23/2018	Class II (510(k))
14	P160007	Guardian Connect System (Medtronic MiniMed)	A continuous glucose monitor with AI assistance.	Clinical Chemistry	03/08/2018	PMA
15	DEN180001	IDx-DR (IDx)	A software program that uses an AI algorithm to analyze retinal images.	Ophthalmic	04/11/2018	Class II (De Novo)
16	K173931	MindMotion GO (MindMaze)	A gamified neurorehabilitation therapy platform using AI.	Physical Medicine	05/17/2018	Class II (510(k))
17	K180455	NeuralBot (Neural Analytics)	A lucid robotic ultrasound system for brain blood flow assessment.	Radiology	05/22/2018	Class II (510(k))
18	DEN180005	OsteoDetect (Imagen Technologies)	A computer-aided detection and diagnostic software that uses an AI algorithm to analyze two-dimensional X-ray images for signs of distal radius fracture.	Radiology	05/24/2018	Class II (De Novo)
19	K173821	LungQ (Thirona Corporation)	A lung quantification software to analyze chest CT scans.	Radiology	06/05/2018	Class II (510(k))
20	DEN170043	DreaMed Advisor Pro (DreaMed Diabetes)	An AI-powered technology to seamlessly treat patients remotely with its virtual diabetes management service.	Clinical Chemistry	06/12/2018	Class II (De Novo)
21	K172983	HealthCCS (Zebra Medical Vision)	An AI-powered software that can be used to evaluate calcified plaques in the coronary arteries.	Radiology	06/13/2018	Class II (510(k))
22	K173780	EchoMD Automated Ejection Fraction Software (Bay Labs)	A system that enables fully automated AI echocardiogram analysis.	Radiology	06/14/2018	Class II (510(k))
23	K180647	BriefCase (Aidoc Medical)	An AI algorithm to detect and triage abnormal findings in non-enhanced head CT images.	Radiology	08/01/2018	Class II (510(k))
24	DEN180042	Irregular Rhythm Notification Feature (Apple)	An application to detect irregular heart rhythms in pulse rate data collected by the Apple Watch photoplethysmograph sensors.	Cardiovascular	09/11/2018	Class II (De Novo)
25	DEN180044	ECG App (Apple)	Applications to detect atrial fibrillations and sinus rhythms in ECG data from an Apple Watch and display the results on an iPhone.	Cardiovascular	09/11/2018	Class II (De Novo)
26	K173872	FibriCheck (Qompium)	A smartphone application for the detection of heart rhythm disorders such as atrial fibrillation.	Cardiovascular	09/28/2018	Class II (510(k))
27	K181771	RightEye Vision System (RightEye)	A cloud-based system that uses objective eye movement measurements to aid in the evaluation of Parkinson’s disease.	Neurology	09/28/2018	Class II (510(k))
28 *	K182034	Arterys MICA (Arterys)	An AI-based platform for analyzing medical images such as MRI and CT.	Radiology	10/17/2018	Class II (510(k))
29	K182177	Accipio Ix (MaxQ-AI)	An AI-enabled software workflow tool that aids in identifying acute intracranial hemorrhage and prioritizing the treatment of strokes or head trauma.	Radiology	10/26/2018	Class II (510(k))
30	K181939	icobrain (icometrix)	A software that extracts clinically meaningful information from brain CT or MRI scans of patients with multiple sclerosis, dementia or brain injury.	Radiology	11/06/2018	Class II (510(k))
31	K180432	AI-ECG Platform (Shenzhen Carewell Electronics)	A software package which is a distributed ECG auto analysis system designed to assist in measuring and interpreting 12-lead resting ECG with an AI algorithm.	Cardiovascular	11/19/2018	Class II (510(k))
32	K182218	FerriSmart Analysis System (Resonance Health Analysis Service)	An automated system for measuring liver iron concentration.	Radiology	11/30/2018	Class II (510(k))
33 *	K182336	SubtlePET (Subtle Medical)	An AI-powered technology that enables centers to deliver a faster and safer patient scanning experience, while enhancing exam throughput and provider profitability.	Radiology	11/30/2018	Class II (510(k))
34	K173681	reSET-O (Pear Therapeutics)	A Prescription Digital Therapeutic (PDT) platform for the treatment of Opioid Use Disorder.	Neurology	12/10/2018	Class II (510(k))
35	K181861	Embrace (Empatica)	An epilepsy smartband that detects patterns in motion and physiological signals that may be associated with generalized tonic-clonic seizures, and immediately alerts caregivers.	Neurology	12/20/2018	Class II (510(k))
36	K182130	iSchemaView RAPID (iSchemaView)	An AI-enhanced advanced medical imaging for stroke.	Radiology	12/27/2018	Class II (510(k))
37	K182564	Quantib ND (Quantib)	An AI solution that helps radiologists read MRI brain scans.	Radiology	12/27/2018	Class II (510(k))
38	K182456	Study Watch (Verily Life Sciences)	A wearable device to record, store, transfer, and display ECG rhythms.	Cardiovascular	01/17/2019	Class II (510(k))
39	K182344	RhythmAnalytics (Biofourmis Singapore)	An AI-powered software to detect irregular heart rhythms when ECG data are uploaded.	Cardiovascular	03/07/2019	Class II (510(k))
40 *	K183285	cmTriage (CureMetrix)	An AI-based triage software for mammography.	Radiology	03/08/2019	Class II (510(k))
41	K181823	KardiaAI (AliveCor)	An AI-based software analysis library to assess ambulatory ECG rhythms from adult subjects.	Cardiovascular	03/11/2019	Class II (510(k))
42	K181352	Loop System (Spry Health)	A tool to collect and transfer physiological data of patients in the home environment.	Cardiovascular	03/29/2019	Class II (510(k))
43 *	K183202	Deep Learning Image Reconstruction (GE Medical Systems)	A deep learning-based CT image reconstruction technology.	Radiology	04/12/2019	Class II (510(k))
44	K181988	eMurmur ID (CSD Labs)	A software screening device that uses a smartphone, electronic stethoscope and machine learning to automate the detection of heart murmurs.	Cardiovascular	04/17/2019	Class II (510(k))
45	K190362	HealthPNX (Zebra Medical Vision)	A radiological computer-assisted triage and notification software system.	Radiology	05/06/2019	Class II (510(k))
46 *	K183046	Aquilion ONE (TSX-305A/6) V8.9 with AiCE (Canon Medical Systems Corporation)	A device to acquire and display cross-sectional volumes of the whole body, including the head, with the capability of imaging whole organs in a single rotation.	Radiology	06/12/2019	Class II (510(k))
47 *	K191384	RayCare 2.3 (RaySearch Laboratories)	An oncology information system used to support workflows, scheduling and clinical information management for oncology care and follow-up.	Radiology	07/08/2019	Class II (510(k))
48	K183322	physIQ Heart Rhythm and Respiratory Module (physIQ)	A device for the calculation of heart rate and heart rate variability, the detection of atrial fibrillation and the determination of respiration rate using ambulatory ECG and triaxial accelerometer data.	Cardiovascular	07/10/2019	Class II (510(k))
49	K191272	Current Wearable Health Monitoring System (Current Health)	A device for reusable bedside, mobile and central multi-parameter, physiologic patient monitoring of adult patients in professional healthcare facilities.	Cardiovascular	07/12/2019	Class II (510(k))
50	K182384	ACR | LAB Urine Analysis Test System (Healthy.io)	A device for the semi-quantitative detection of albumin and creatinine in urine, using a smartphone application, a proprietary Color-Board, and ACR Reagent Strips.	Clinical Chemistry	07/26/2019	Class II (510(k))
51	K183271	AI-Rad Companion (Pulmonary) (Siemens Medical Solutions USA)	An image processing software that provides a quantitative and qualitative analysis from previously acquired CT DICOM images to support radiologists and physicians in the evaluation and assessment of lung disease.	Radiology	07/26/2019	Class II (510(k))
52	K183282	Biovitals Analytics Engine (Biofourmis Singapore)	An AI-based software engine used with continuous biometric data from already cleared sensors measuring heart rate, respiratory rate, and activity in ambulatory patients being monitored in a healthcare facility or at home, during periods of minimal activity.	Cardiovascular	08/15/2019	Class II (510(k))
53	K183268	AI-Rad Companion (Cardiovascular) (Siemens Medical Solutions USA)	An image processing software that provides quantitative and qualitative analysis from previously acquired CT DICOM images to support radiologists and physicians in the evaluation and assessment of cardiovascular diseases.	Radiology	09/10/2019	Class II (510(k))
54	K190815	BrainScope TBI (BrainScope Company)	A portable, non-invasive, non-radiation emitting, point of care device intended to provide results and measures to support clinical assessments and aid in the diagnosis of concussion/mild traumatic brain injury (mTBI).	Neurology	09/11/2019	Class II (510(k))
55	K191688	SubtleMR (Subtle Medical)	An image processing software that can be used for image enhancement in MRI images.	Radiology	09/16/2019	Class II (510(k))
56 *	K191994	ProFound AI Software V2.1 (iCAD)	A CAD software device intended to be used concurrently by interpreting physicians while reading digital breast tomosynthesis (DBT) exams from compatible DBT systems.	Radiology	10/04/2019	Class II (510(k))
57	K191713	CS-series-FP Radiographic/Fluoroscopic Systems with Optional CA-100S/FluoroShield (Omega Medical Imaging)	A modification device to provide an automated region of interest that reduces exposure to the patient and operator.	Radiology	10/04/2019	Class II (510(k))
58	K191171	EchoGo Core (Ultromics)	A software for use in quantification and reporting of results of cardiovascular function to support physician diagnosis.	Radiology	11/13/2019	Class II (510(k))
59 *	K192287	Transpara (ScreenPoint Medical)	A device for use as a concurrent reading aid for physicians interpreting screening mammograms from compatible FFDM systems to identify regions suspicious for breast cancer and assess their likelihood of malignancy.	Radiology	12/10/2019	Class II (510(k))
60	K192004	Eko Analysis Software (Eko Devices)	A software to provide support to the physician in the evaluation of patients’ heart sounds and ECG’s.	Cardiovascular	01/15/2020	Class II (510(k))
61	DEN190040	Caption Guidance (Caption Health)	A software to assist in the acquisition of cardiac ultrasound images.	Radiology	02/07/2020	Class II (De Novo)

*: The product involved in the field of oncology. Abbreviations: AI, artificial intelligence; US FDA, United States Food and Drug Administration; CT, computed tomography; MRI, magnetic resonance imaging; DICOM, Digital Imaging and Communications in Medicine; ECG, electrocardiogram; FFDM, full-field digital mammography; ADHD, attention deficit hyperactivity disorder.
